# In Situ Observations Reveal Underestimated Greenhouse Gas Emissions from Wastewater Treatment with Anaerobic Digestion – Sludge Was a Major Source for Both CH_4_ and N_2_O

**DOI:** 10.1021/acs.est.5c04780

**Published:** 2025-08-22

**Authors:** Magnus Gålfalk, David Bastviken

**Affiliations:** Department of Thematic Studies − Environmental Change, Linköping University, Linköping 581 83, Sweden

**Keywords:** methane, nitrous oxide, wastewater treatment, anaerobic sludge, drone, UAS, emission factors

## Abstract

Wastewater treatment plants (WWTPs) receive large and increasing organic carbon and nitrogen flows through societies. Consequently, WWTPs emit greenhouse gases (GHGs), including methane (CH_4_) and nitrous oxide (N_2_O). However, large uncertainties remain, as direct measurements of WWTP emissions have been challenging, and emission estimates frequently depend on uncertain emission factors and activity data. Using drone-based measurements, we here show that the combined CO_2_-equivalent emissions of CH_4_ and N_2_O from WWTPs with anaerobic digestion (AD) and sludge storage were 2.4-fold higher than IPCC-recommended emission-factor-based estimates. N_2_O emissions from sludge, presently assumed to be zero, were 9% of the CH_4_ emissions by weight and contributed to half of the total CO_2_-equivalent sludge emissions. Hence, with the necessary increase in AD in WWTPs to recover energy and reduce fossil fuel use, emission mitigation is needed, and adequate tools facilitating flux observations by WWTP managers are key for setting effective mitigation priorities.

## Graphical abstract

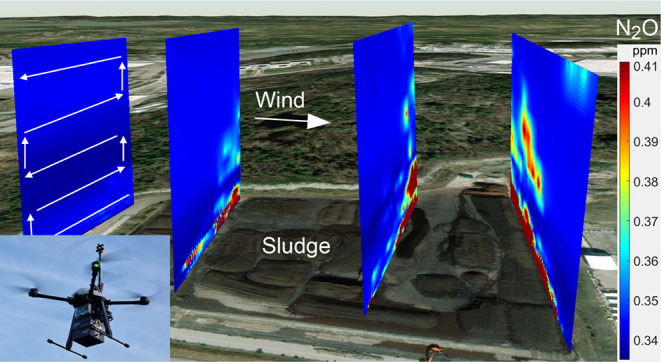

## Introduction

An increasing share of global households (hitherto estimated at 44–52%) and industries deliver organic waste to WWTPs.^1,2^ This represents one of the largest global flows of nitrogen-rich organic matter through society. However, greenhouse gas (GHG) emissions from WWTPs are highly uncertain. Comprehensive in situ flux observations are scarce, and the risk of bias in nationally reported emission assessments has been highlighted.^3–6^

WWTPs are complex environments with many processes and treatment steps, from which representative GHG fluxes are challenging to measure. Most GHG emission estimates from WWTPs are based on the IPCC guidelines for national GHG inventories via emission factors (EFs).^7,8^ The EFs are based on a combination of existing emission observations, theoretical constraints on the maximum amounts of GHG formation given the organic matter (OM) load, and expert opinions. Some countries have generated Tier 2 or Tier 3 EFs (including more specific information than the basic Tier 1 EFs) based on national data, which is encouraged in the IPCC guidelines. Activity data are used to scale emissions to local conditions^8^ and at the Tier 1 level, activity data can include the number of person-equivalents connected to a WWTP, the biological or chemical oxygen demand (BOD or COD, respectively), and the total nitrogen (*N*_tot_) content of the material in the plant. There are also standardized correction factors for differences among countries and types of WWTP processes.

The EF-based approach has yielded global CH_4_ emission estimates from WWTPs of 14–33 Tg CH_4_ yr^–1^, corresponding to 2.5–6% of the total CH_4_ emissions, or 4–9% of anthropogenic emissions.^9^ These emissions are significant and important to study, as they are anthropogenic, with a high potential for mitigation or gas collection compared to natural emissions. Similarly, global WWTP N_2_O emissions have been estimated at 0.16 (min 0.02 to max 0.73) Tg N_2_O yr^–1^, representing 1% or 2.5% of the global total and anthropogenic N_2_O emissions, respectively.^10,11^ The carbon dioxide (CO_2_) emitted from the waste material is considered renewable because it is derived from recently produced biomass, while CH_4_ and N_2_O formed in the WWTP treatments are considered anthropogenic additions to atmospheric GHG emissions.

The present emission-factor-based estimates have placed WWTPs among the minor emission sources, releasing <5% of the total global emissions of the respective GHGs. However, the estimated CH_4_ and N_2_O emissions from WWTPs have been challenged. Some studies have reported that IPCC EFs exceeded observation-based EFs.^3,5,12^ However, most in situ observations reported are based on specific treatment steps, making them difficult to compare with full-WWTP emissions. To date, the seemingly most comprehensive in situ observations of CH_4_ fluxes from full WWTPs used the tracer gas methodology and indicate that EFs are considerably underestimated.^13^ Similarly, in situ observations of emissions from mixed types of full-scale separate AD facilities (e.g., biogas production plants) using the same methodology also indicate substantially underestimated CH_4_ emission factors.^6^ In addition, in situ measurements with hyperspectral infrared spectroscopy discovered new WWTP fluxes typically not considered in EF-based frameworks, such as large CH_4_ emissions from ineffective flaring^14^ adding reasons for the need for effective in situ observations.

Potentially large emissions of N_2_O from the nitrogen treatment steps are well-known and have received considerable attention (e.g., ref^15^). N_2_O emissions from dewatered AD sludge have been mentioned as potentially significant,^16^ but the IPCC guidelines on GHG emission estimation explicitly state that these emissions are negligible^7,8^ and in situ measurements of such fluxes are rare. Yet, the consideration of N_2_O emissions is important, as the N_2_O global warming potential is ∼300 times that of CO_2_, and mitigating N_2_O emissions could also reduce undesirable loss of sludge nitrogen that would be better used in farmland fertilization.

The assumption of zero N_2_O emission from AD sludge is in contrast with the well-known N_2_O emissions from compost.^17,18^ AD sludge, after leaving the digestion chamber and being exposed to air during storage, will develop conditions similar to those of compost in the exposed surface layers. The stored sludge is nitrogen-rich and will develop a surface oxygen gradient that should fluctuate in thickness depending on weather (wind and precipitation). Therefore, AD sludge piles are likely to behave as compost in terms of N_2_O production and emissions during outdoor storage,^16^ and if this is correct, considerable N_2_O emissions are to be expected also from anaerobic sludge.

With the aim of comparing emission factor estimates and in situ measurements for multiple WWTPs, the underlying hypotheses were (i) that AD sludge piles emit significant amounts of N_2_O in contrast to common assumptions, and (ii) that comprehensive in situ field-scale observations would show greater combined emissions of CH_4_ and N_2_O than expected from emission factor estimates.

Informed by our past detailed hyperspectral mapping of CH_4_ and N_2_O emissions from one WWTP, revealing the importance of multigas large-scale assessments,^14,19^ we have here used a recently developed drone-based approach^20^ to quantify both CH_4_ and N_2_O emissions from selected high-emission treatment steps at 13 WWTP facilities (12 full plants and one large sludge storage facility serving multiple additional WWTPs). The studied WWTPs used the common Swedish treatment setup with AD for energy and nutrient recovery and for minimizing the fossil fuel footprint^21^ (for more details on WWTP and process design, see the Supporting Information). Such treatment designs are frequently used in large WWTPs,^13^ and the rapid ongoing WWTP expansion,^2^ combined with the need to reduce fossil fuel use and to recover energy from waste, will likely lead to increased use of AD in WWTPs.^21,22^ To our knowledge, this type of multi-WWTP in situ assessment of combined CH_4_ and N_2_O emissions from AD sludge, with coverage of full-scale sludge piles, has not been possible prior to the development of the drone-based method we used.^20^

## Materials and Methods

### Wastewater Treatment Plants

This study focuses on the direct gas-specific emissions of CH_4_ and N_2_O from WWTPs, motivated by the apparent knowledge gaps on these emissions (given, e.g., that full-scale measurements of sludge piles are rare for CH_4_ and not conducted for N_2_O), in turn preventing clarity on which mitigation efforts would be most effective. To put the measurements in context, a brief overview of wastewater treatment strategies and setups where the largest emissions are expected is provided before the regular methods description.

Key aims of wastewater treatment are to quickly remove organic material (OM; often measured as biological oxygen demand, BOD), phosphorus (P), nitrogen (N), and sometimes also other pollutants before releasing treated water into the recipient water. A common strategy for WWTPs with AD includes the following steps:

Step 1 Mechanical grit removal.
Step 2 Chemical flocculation of e.g., P and some OM. The flocculated particles are transferred to steps 6 and 7, and the remaining water is sent to step 3.
Step 3 Biological treatments are used to remove OM by microbial degradation and to convert ammonia to nitrate (oxic treatment) and then to nitrogen gas via denitrification (anoxic treatment).
Step 4 Harvesting of particles after step 3, which are mostly directed to steps 6 and 7 and some are rerouted to step 3 as microbial inoculum. The remaining water is transferred to step 5.
Step 5 Polishing of the water before release to downstream recipient water. This can include tertiary treatments such as advanced oxygenation processes, filtration, adsorption, ion exchange, disinfection, or passage through polishing ponds or wetlands.
Step 6 Particulate matter harvested in the above steps is gathered as sludge. The sludge can be dewatered, with the water being sent back to a suitable previous step. Harvesting of energy and nutrients from the sludge is a major goal toward circular economies, often leading to AD of the sludge to recover bioenergy as CH_4_, as well as nutrients in the residual sludge after the digestion, ideally for fertilization of farmland if contaminant levels are low enough.
Step 7 After AD, postdigestion long-term sludge storage for one to six months is common to reduce the levels of pathogens before using the sludge residual. This is often done as a pile for each month.

The text above is a general description of a common WWTP setup with anaerobic digestion. There are other alternatives, and for each alternative, many detailed options for each process exist, as reviewed elsewhere (e.g., ref^10^).

The largest CH_4_ emissions from the WWTP described above with AD have primarily been associated with step 7 (sludge storage). Other locations with expected CH_4_ leakage are the anoxic environments, e.g., in step 3, and also emissions from anaerobic degradation of released OM in the effluent reaching recipient waters (e.g., late phases of step 5 and beyond; included in the IPCC Tier 1 estimations). The large amounts of CH_4_ produced in step 6 are not emitted but harvested and used as biogas. N_2_O emissions have previously primarily been associated with steps 3 and 4 (oxic parts) and emissions from the effluent water, and, as noted, no emissions at all have been expected from step 7 (AD sludge storage).^8^

We focused on measuring emissions from sludge storage, the steps for N and P removal, and the biological treatment, as these had been shown in a previous study to be the highest emitters of CH_4_ and N_2_O, respectively.^14^ The 12 WWTPs selected for this study (Table 1) represented a large variation in influent wastewater, the number of person equivalents (pe) connected (from 42 600 to 1 000 000 pe), methods used for nitrogen removal, and average sludge storage time. These WWTPS are distributed across southern Sweden. We also included a larger sludge management facility that receives and stores sludge from multiple WWTPs. Our first campaign took place from March to July 2022 with separate flights for CH_4_ and N_2_O. The second campaign, using a drone with the ability to lift more instrumentation, measured both CH_4_ and N_2_O simultaneously and was carried out from June to August 2023 and in April 2024. Many of the WWTPs were included upon request from operations managers, which signals widespread engagement and interest in gaining increased emission knowledge and in mitigating the emissions at the WWTPs.

**Table 1:** WWTP Background Information and Summary of Results^d^ ^e^ ^f^

Background information														
WWTP	1	2	3	4	5	6	7	8	9	10	11	12	13	**Avg.**
pe (thousands)	42.6	110.0	70.0	446.1	81.2	94.1	197.6	154.0	824.5	76.7	33.0	198.0	1007.6	**256.6**
BOD in (t yr^–1^)	1600	2851	1331	11 397	2077	2405	4887	5403	22 090	1960	715	4090	20 082	**6222**
BOD out (t yr^–1^)	20	33	12	498	47	17	42	30	906	27	13	65	579	**176**
*N*_tot_ in (t yr^–1^)	320	412	291	1845	448	529	776	795	3852	340	143	930	---	**890**
*N*_tot_ out (t yr^–1^)	69	65	153	562	87	83	49	60	976	87	47	180	1291	**285**
Sludge production (t yr^–1^)	5170	8146	6600	26 978	5091	6320	9102	10 800	51 924	5148	3312	14 028	67 560	**16 937**
IPCC (Tier 1)														
CH_4_-T1 (t yr^–1^)	4.56	7.21	5.53	32.03	5.06	5.41	8.16	9.27	60.57	4.69	2.93	12.59	66.22	**17.25**
N_2_O-T1 (t yr^–1^)	2.28	2.15	5.05	18.54	2.87	2.74	1.62	1.98	32.21	2.88	1.56	5.94	42.59	**9.41**
IPCC Swedish (Tier 2)														
CH_4_-T2 (t yr^–1^)	6.10	10.87	7.30	43.44	7.92	9.17	20.62	20.59	84.19	7.47	2.73	15.59	76.54	**24.04**
N_2_O-T2 (t yr^–1^)	2.17	2.04	4.81	17.66	2.73	2.61	1.54	1.89	30.67	2.74	1.48	5.66	40.56	**8.97**
Measurements (M)														
CH_4_-M (t yr^–1^)	3.68	12.45	47.09	116.44	28.36	16.75	43.07	76.65	471.03	62.43	20.52	144.07	319.05	**104.74**
CH_4_-M sludge fraction	osm	osm	osm	0.84	0.81	nsm	osm	osm	0.74	0.82	0.74	0.73	osm	**0.78**
N_2_O-M (t yr^–1^)^a^	5.97^b^	7.79^b^	6.38^b^	16.14^b^	2.61^b^	19.60	6.88	7.58^b^	68.28	13.05^b^	5.43	11.91	26.02	**15.20**
N_2_O-M sludge fraction	0.05^b^	0.14^b^	0.63^b^	0.52^b^	0.75^b^	nsm	0.43	0.87^b^	0.45	0.34^b^	0.36	0.57	osm	**0.47**
Ratios of measured (M) versus EF-estimated (T)														
CH_4_-M/CH_4_-T1	0.81	1.73	8.51	3.64	5.61	3.10	5.28	8.27	7.78	13.32	7.01	11.45	4.82	**6.25**
CH_4_-M/CH_4_-T2	0.60	1.15	6.45	2.68	3.58	1.83	2.09	3.72	5.59	8.36	7.53	9.24	4.17	**4.38**
N_2_O-M/N2O-T1	2.62^b^	3.63^b^	1.26^b^	0.87^b^	0.91^b^	7.16	4.26	3.83^b^	2.12	4.53^b^	3.49^c^	2.01	0.61^c^	**2.87**
N_2_O-M/N2O-T2	2.75^b^	3.81^b^	1.33^b^	0.91^b^	0.95^b^	7.51	4.47	4.02^b^	2.23	4.76^b^	3.67^c^	2.11	0.64^c^	**3.01**
CO_2_eq-M/CO_2_eq-T1	2.32	3.15	1.97	1.27	1.61	6.49	4.60	5.23	3.01	5.75	4.04	3.64	1.19	**3.41**
CO_2_eq-M/CO_2_eq-T2	2.29	2.89	2.00	1.26	1.54	6.05	3.11	3.86	2.95	5.52	4.26	3.63	1.21	**3.12**

a Yearly emissions are representative of the sludge age distribution of the piles at each WWTP (which is kept constant).

b Sludge N_2_O emissions were estimated using the average N_2_O/CH_4_ sludge emission ratio of WWTPs 7, 9, 11, 12, and 13 (average ± SD was 0.086 ± 0.029 kg N_2_O per kg CH_4_ emitted).

c Underestimates. Measurements (M) of sludge emissions only while T-estimates assume sludge N_2_O emissions to be zero. Accordingly, the mean values in the rightmost column are also underestimated.

d WWTPs are numbered. Pe is person-equivalents. BOD and N_tot_ are biological oxygen demand and total nitrogen, respectively. T1 and T2 denote emission estimates based on IPCC Tier 1 guidelines and Swedish Tier 2 methodology, respectively. M denotes our measurements (see the Methods for details). Nitrogen variables are shaded gray. CO_2_eq represents 100-yr CO_2_ equivalents (using mass-based conversion factors of 27 for CH_4_ and 273 for N_2_O).^25^

e nsm: no sludge emissions measured; hence, total CH_4_ and N_2_O estimates are conservative.

f osm: only sludge emissions measured; hence, CH_4_ and N_2_O estimates are conservative.

### Drone Systems and Flux Method

We have used two uncrewed aircraft systems (UASs), having all necessary equipment on a drone (sensors for CH_4_, N_2_O, temperature, pressure, relative humidity, and an anemometer for wind, 3D positioning and other accessory variables). This setup makes the airborne measurements fully independent from other data sources – as hence referred to as iUAS. With the iUAS, it is thereby possible to simultaneously measure GHG concentrations and air movements at high frequency along a flight path. Repeated flight tracks along virtual walls surrounding target areas allowed for mass-balance calculations, subtracting downwind from upwind gas transport (export minus import) to calculate mean fluxes from the target areas. These areas can have different sizes, e.g., from approximately 10 × 10 m^2^ to hectare-sized plots. This approach was previously developed for CH_4_ ^20^ and was here further developed to measure CH_4_ and N_2_O simultaneously (Figures 1–3, S3, and S4).

**Figure 1:**
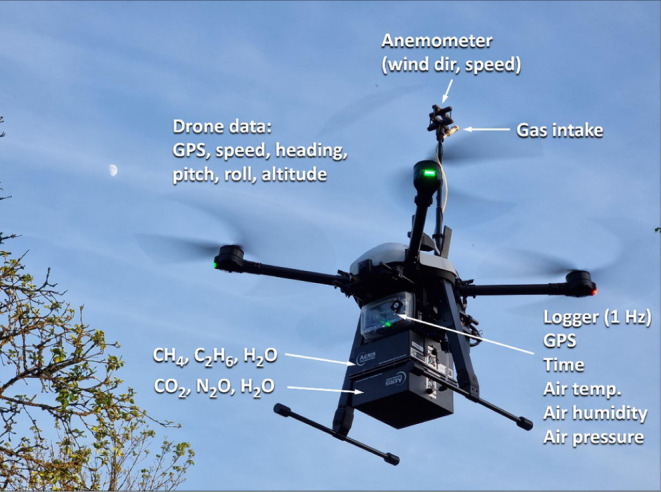
An iUAS was used for simultaneous measurements of GHGs, telemetry, and meteorology. The image shows system 2 as described in Methods (iUAS-2).

**2:**
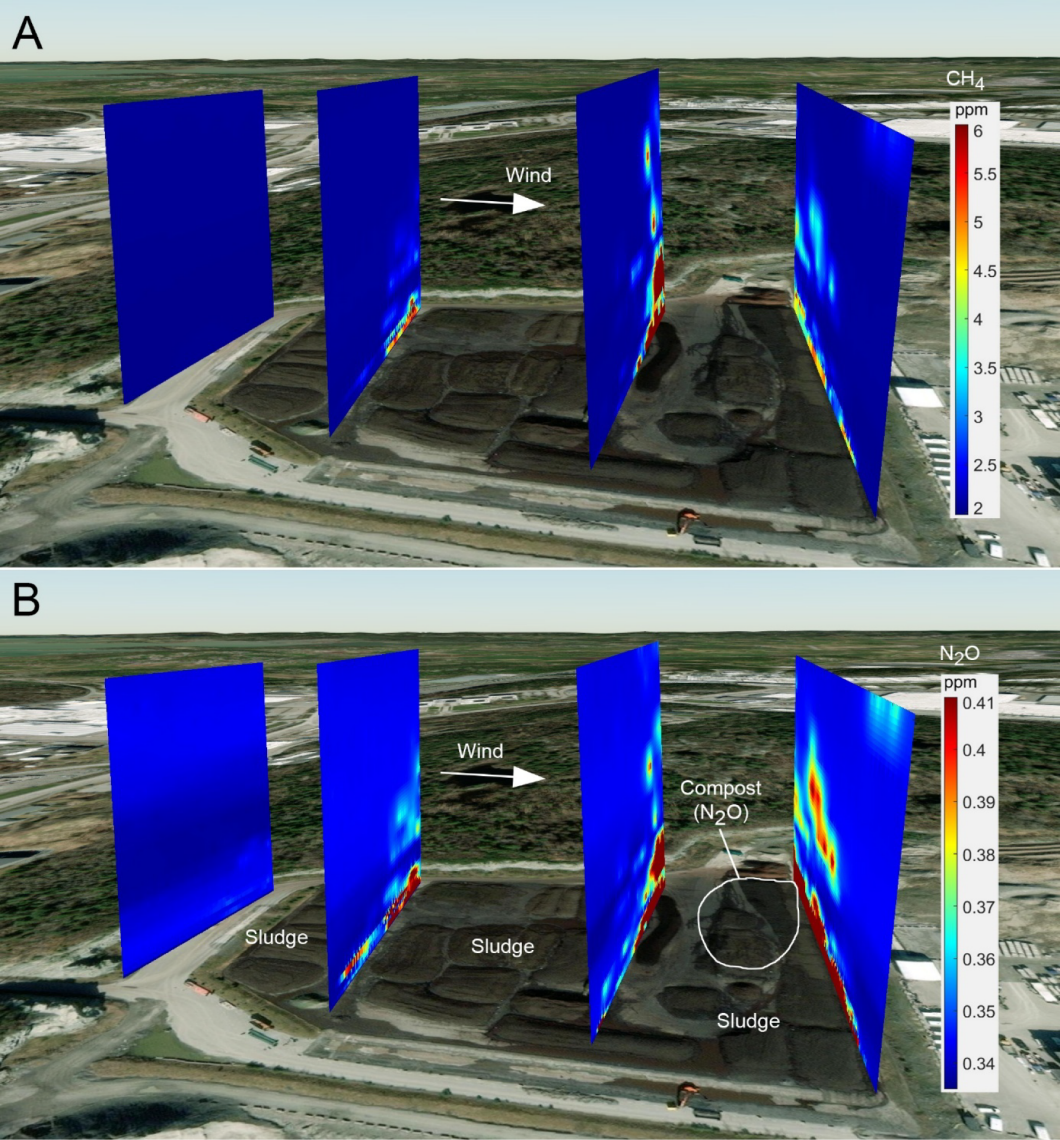
Vertical gas concentration maps at a sludge storage site. These walls were flown perpendicular to the dominant wind direction and are shown for CH_4_ (A) and N_2_O (B) at one of the studied WWTPs. Simultaneous measurements of concentrations, wind speed, and wind direction at 1 Hz during the flights allow the calculation of mass transport across each wall. The difference between incoming (upwind) and outgoing (downwind) wall mass transport allows the assessment of emissions. The four vertical wall flights (70, 80, 90, and 100 m high) enable the sludge storage site to be divided into three sections. Higher N_2_O emissions are seen across the rightmost wall due to compost material, as indicated. Note that the figure shows gas concentration fields, while the total gas transport is also related to air transport (wind speed). Hence, higher concentrations combined with lower wind speed can represent similar mass transport as low concentrations combined with greater wind speeds (see the Supporting Information for an example). The background image was created using WebMap’s World Imagery in MATLAB.

**Figure 3:**
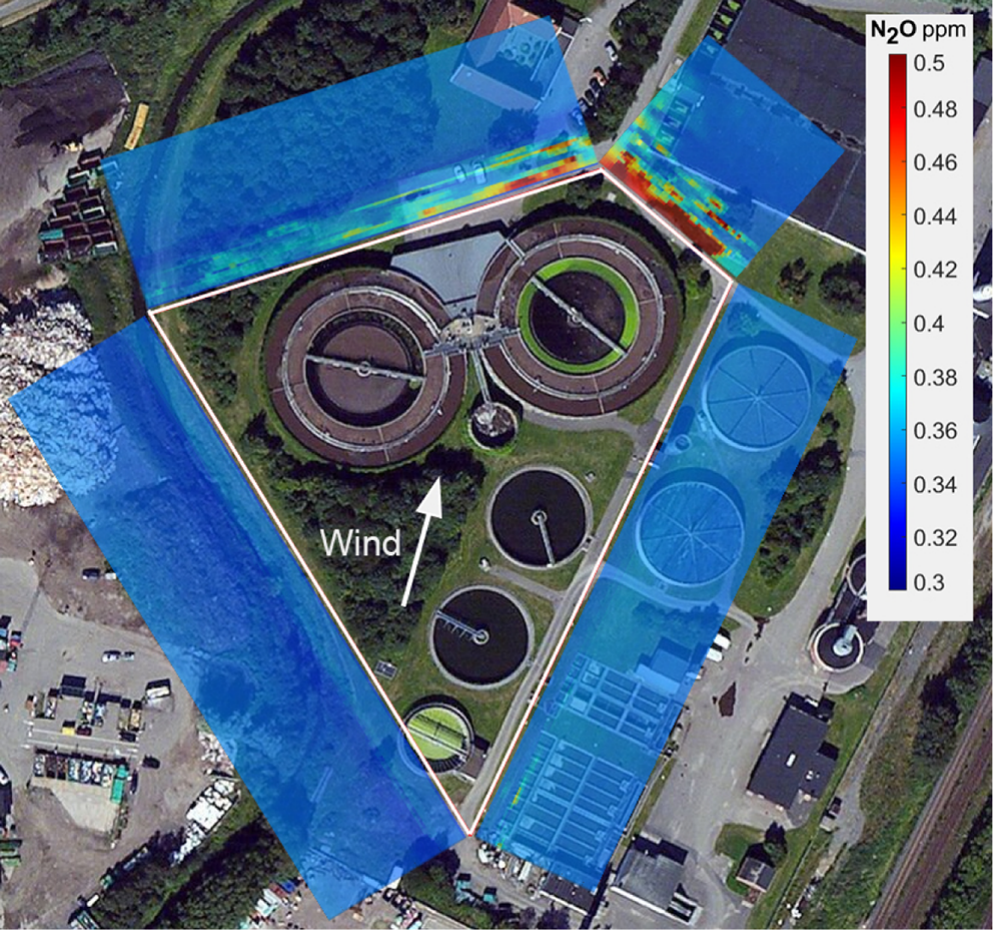
Vertical iUAS flight walls with colors representing the N_2_O concentration around WWTP nitrogen treatment tanks. The walls (55 m high) were vertical but are tilted outward in the image for visual clarity. The N_2_O mass transport into and out of the encircled area allows emission assessments. The downwind walls clearly show N_2_O emission from the area.

The first iUAS-system used in this study (referred to as iUAS-1) was a DJI Matrice 300 drone, equipped with either an Aeris Miro Pico CH_4_ or an Aeris Strato N_2_O sensor mounted (each weighing 1.9 kg; the maximum payload is 2.3 kg), a Trisonica anemometer placed above the propellers on a carbon fiber rod, smaller sensors for air pressure, air temperature, relative humidity, and a logger that stored all the measurements at 1 Hz (5 Hz for wind measurements), including drone telemetry such as heading, speed, altitude, and GPS coordinates. This system had a flight time of 30 min but is otherwise similar to the iUAS system presented in Gålfalk et al.^20^ The second system (iUAS-2; Figure 1) was a larger Airolit Explorian XLT drone with a maximum payload of 5 kg, allowing both an Aeris Strato CH_4_/C_2_H_6_ and an Aeris Strato N_2_O/CO_2_ sensor to be flown simultaneously. This system also carried a Trisonica anemometer, a logger, and the same accessory sensors as on iUAS-1. Wind measurements were verified against separate ground-based wind observations (Vaisala WXT520) within 15 m in open terrain to confirm negligible bias from the drone.^20^ The gas intake was positioned near the anemometer (Figure 1).

The mass balance method was used to obtain fluxes by flying back and forth at different altitudes to create virtual vertical walls (Figure S2), sampling the upwind (incoming) and downwind (outgoing) air of a target area;^23^ details are provided in Gålfalk et al.^20^ Flights were conducted as close to the ground as possible (about 1 to 1.5 m) and up to a maximum of 120 m when necessary to sample the full plume from a treatment step or WWTP area. Gas concentration and wind speed maps (perpendicular to the average wind direction) for each flight wall were used for mass balance calculations. Flight speeds were maintained at about 3 m s^–1^ as a compromise between flight time and allowing sampling at a high enough spatial resolution (one sample every third meter). In the case of a small target area (e.g., an individual sludge pile), the flight speed was maintained at about 1 m s^–1^ to obtain a 1 m spatial resolution.

iUAS-2 used an external battery to run the two gas sensors in preparation for flights before powering the drone on and during battery changes, as an improved strategy for reducing the startup time, as the sensors do need several minutes to stabilize before starting measurements. Altitude was primarily read from the drone GPS position and was also, in some cases, verified by altitude calculations from logged air pressure measurements. All parameters were stored together in a log file at 1 Hz, except for wind speed and direction, which were stored at 5 Hz (and were resampled to average values at 1 Hz). The delay between gas intake and measurement time was calculated using a breath test, where air was blown close to the intake tube, and the delay in time until the corresponding peak of the CO_2_ and CH_4_ concentrations was measured; this was found to be 8 s for iUAS-1 and 5.5 s for iUAS-2.

In the first campaign with iUAS-1, only one sensor could be used per flight, and we prioritized the CH_4_ sensor when measuring sludge storage, as N_2_O emissions from sludge have been assumed to be negligible in the IPCC guidelines.^7,8^ When using iUAS-2 in the second campaign, the high sludge N_2_O emissions were discovered, and the mean ratio of N_2_O to CH_4_ emissions from simultaneous measurements in campaign 2 was used to estimate sludge N_2_O emissions in campaign 1.

The measurements were designed to cover the greatest emission sources based on local treatment process knowledge and previous experiences^24^ in dialogues with WWTP managers. Priority was given to sludge treatment, sludge storage, and treatment steps involving nitrogen removal. Hence, measurements did not include all possible emissions from the WWTPs, and results should be seen as minimum estimates that underestimate the real total emissions. This makes the reported fluxes conservative (and adds robustness to the main conclusion).

The flux density (mass of target gas passing an area per time unit) for each 1 Hz measurement point across the virtual flight-walls (*F*_gp_; “g” denotes the gas in focus; “p” denotes individual measurement points) was calculated according to

1 $$F_{\mathrm{gp}}=\left(n_{g}{/V}_{\mathrm{air}}\right)\times M_{g}\times v_{\mathrm{wp}}$$

where

*n*_g_ is the amount of target gas (mol).
*V*_air_ is the air volume (m^3^).
(*n*_g_/*V*_air_) = *p*_g_/*RT* (yields the target gas concentration given by the common gas law; mol/m^3^; *p*_g_ is the partial pressure of the gas in focus, yielded by dividing the parts-per-billion output from the Aeris gas analyzers with 10^9^ and multiplying by the barometric pressure; *R* is the common gas constant with compatible units; *T* is the temperature).
*M*_g_ is the molar mass of the target gas (g/mol).
*v*_wp_ is the windspeed component perpendicular to the virtual wall (m/s; plus or minus signs define directions relative to the wall, thereby accounting for wind variability during the flights).

This can be rewritten as follows using parameters that are measured by the drone:

2 $$F_{g}\left(x,y\right)=\left(M_{g}/R\right)\times \left(P_{\mathrm{air}}{/T}_{\mathrm{air}}\right)\times X_{g}\left(x,y\right)\times {\mathrm{10}}^{-6}\times v_{\mathrm{wp}}\left(x,y\right)$$

where

*x* and *y* are the coordinates on the wall (m).
*P*_air_ and *T*_air_ are the air pressure (Pa) and air temperature (K), respectively.
*X*_g_ is the gas concentration (ppm).
*M*_g_/*R* is an easy-to-look-up constant that depends on the gas species.
*F*_g_ is measured in g/m^2^/s.

After setting up a flux matrix with 1 × 1 m resolution for each wall, all relevant points of the measured point cloud (being part of that wall) were interpolated onto the matrix at sub-m resolution. After gap-filling using spring interpolation, the flux density across each virtual wall (*F*_g_) was calculated by integrating over the flux matrix. Similar matrices were made for perpendicular wind speed and gas concentrations for each species. When the target area was boxed, net import to and export from the target area via all walls were accounted for. When flight conditions favored measurements along upwind and downwind walls, the net difference between *F*_g_ among walls along the net wind direction represented an estimate of the gas contribution or uptake between the walls. Care was taken to plan flight paths relative to wind directions and to select only parts of the walls relevant to the target area after examining the concentration fields, to avoid contamination from other nearby sources.

The fluxes presented in this article are average fluxes during the measurement period, which is often 20–40 min (one or two batteries), depending on the number of walls that need to be flown to encompass the treatment step for a given average wind direction. The uncertainty ranges were calculated using the Monte Carlo method (100 models) to randomly perturb wind measurements (direction and speed) using the measured wind variability (obtained from the variation between nearby points in the point cloud). The uncertainty in gas concentrations is also included in these models, and the total uncertainty for a flux measurement is then calculated from the standard deviation of the total flux from all the simulated perturbations.

Additional drone-method discussion and a general overview of methods for WWTP GHG emission measurements are provided in the Supporting Information.

### Calculation of Emission Factors (Tiers 1 and 2)

We have calculated Tier 1 emission factors for all the WWTPs in the study using IPCC guidelines^7,8^ assuming no composting, as all the WWTPs have anaerobic digestion with biogas production and on-site storage of sludge (assumed to be on a wet-weight basis). N_2_O emissions in these calculations are related to the aquatic treatment steps at a WWTP, in combination with nitrogen in the effluent water from a WWTP. For the Tier 2 emission factors, we have used the Sweden-specific calculations given by the Swedish Environmental Protection Agency.^25^ For both Tier 1 and Tier 2, N_2_O emissions from anaerobic digestion of sludge are given as 0 (as they are assumed to be insignificant).

## Results and Discussion

A summary of the results is shown in Table 1. Total measured CH_4_ emissions from the different WWTPs were in the range of 3.7–471 t CH_4_ yr^–1^, with 78% on average coming from sludge storage. The corresponding total N_2_O emissions were 1.9–68.3 t N_2_O yr^–1^, with 47% coming from sludge storage. The large span in emissions between the WWTPs is due to different solutions for sludge storage, with some WWTPs only having one sludge pile stored on-site for a couple of weeks, while others store sludge on-site in a new pile for each month, up to a year. This makes our total emission calculations for sludge represent lower limits, as emissions will continue at external storage sites outside of the WWTPs in cases of short storage times. Emissions of N_2_O from AD sludge were therefore far from negligible, as commonly assumed.^7,8^ Instead N_2_O emissions from the AD sludge storage were extensive everywhere measured (five WWTPs), corresponding to 8.6 ± 2.5% by mass of the sludge CH_4_ emission. N_2_O contributed 46 ± 7% (mean ± SD) of the 100-year CO_2_ equivalent (CO_2eq_) emission from sludge storage in the studied WWTPs.

Measured emissions were greater than both IPCC Tier 1 and Swedish Tier 2 emission estimates in all but one and three WWTPs for CH_4_ and N_2_O, respectively (Table 1). On average, our observation-based emission estimates were 4.4–6.3, 2.9–3.0, and 3.1–3.4-fold greater for CH_4_, N_2_O, and CO_2eq_, respectively (Table 1). Overall, total N_2_O emissions were 8–160% of those of CH_4_ emissions by mass (median 14%) and dominated the total measured CO_2eq_ emissions (45–95%; mean ± SD of 65 ± 18%; median 59%). Given these results, including the unexpected but consistent and large observed N_2_O emission from sludge storage, N_2_O emissions from WWTPs appear to be at least as important as CH_4_ emissions for global warming.

Using our current and previous data^14,20^ on sludge storage, it is clear that, at least for air temperatures above 5 °C CH_4_ emissions are much more influenced by sludge age than by outside temperature (Figure 4). For calculating yearly CH_4_ emissions, we therefore assume, as a lower estimate, an average flux for the temperature range of 5–30 °C and no flux below 5 °C. For Sweden, most large WWTPs are in a climate ranging from Stockholm (62.9% of daily avg. temperatures above 5 °C over the last 10 years) to Malmö 73.5% daily avg. temperatures above 5 °C over the last 10 years). Even with these conservative assumptions, the actual fluxes would be a factor of 3.3 and 2.4 higher than the IPCC-recommended Tier 2 emission factor for CH_4_ and CO_2eq_, respectively. On top of this, we also note that in a future warmer climate, a greater proportion of days will have temperatures above 5 °C, likely leading to increased sludge emissions.

**Figure 4:**
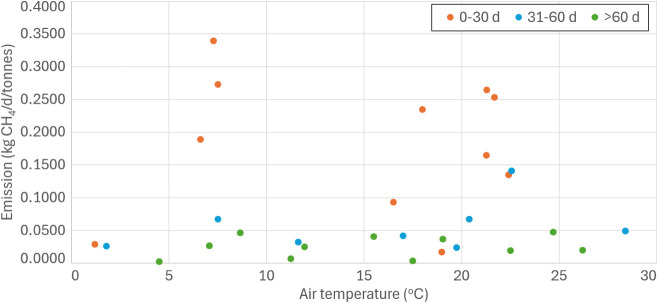
Sludge storage CH_4_ emissions with air temperature and sludge age. The size of emissions is much more dependent on sludge age than air temperature (at least down to 5 °C). The data used are from this publication: Gålfalk et al. 2021 ^20^ (UAV) and Gålfalk et al. 2022 ^14^ (Hyperspectral Imaging) covering the months Jan–Nov.

Our results suggest that GHG emissions from WWTPs with anaerobic digestion are 2.9-to-6.3-fold greater (2.4-to-4.7-fold on a yearly basis) than expected from national inventory estimates, depending on the gas and Tier level compared. Likely, these values are underestimated as not all emissions were covered; Table 1. These results are supported by some of the most ambitious in situ observations of full-plant WWTP CH_4_ emissions by mobile on-ground measurements.^13^ Their work, including 63 plants both with and without AD, indicates that real WWTP CH_4_ emissions are 1.5 to 2.4-fold greater than estimates based on emission factors. Figure S5 shows a comparison of CH_4_ emissions from WWTPs in this and other previous studies. We found no previous in situ measurements of N_2_O emissions at scales comparable to our measurements. However, our results are within the range of N_2_O emissions from small-scale measurements (the studies involving wastewater treatment in Table 2) and were consistent among WWTPs (Table S2). This means that emissions of N_2_O from dewatered sewage AD sludge are likely among the largest 100-yr-CO_2eq_ emission sources from WWTPs (Table 1). Past flux assessments that ignored such emissions have most likely yielded underestimated fluxes.

**Table 2:** A Summary of Studies Where N_2_O Emissions Have Been Measured from Anaerobic Digestion

Reference	Method^a^	Type	Open/closed measurement volume	No. of facilities studied
Colón et al. 2012 ^28^	In-situ (point)	Solid waste	Closed	1
Maulini-Duran et al. 2013 ^29^	Lab (point)	Wastewater	Closed	1
Beylot et al. 2015 ^30^	In-situ (point)	Solid waste	Closed	1
Willén et al. 2016 ^16^	Lab (chamber)	Wastewater	Closed/Open	2
Zeng et al. 2016 ^31^	Lab (point)	Solid waste	Closed	1
Li et al. 2018 ^32^	Lab (point)	Biowaste	Closed	1
Preble et al. 2020 ^33^	In-situ (point)	Solid waste	Closed	1
This study	In-situ (cloud)	Wastewater	Open	6

a Method includes location (in situ and Lab being outside and inside, respectively) and, within parentheses, the type of sample (point, chamber volume, or a point cloud).

The release of N_2_O from nitrogen removal systems in WWTPs is well-known and has been quantified in several previous studies.^26,27^ The nitrogen treatments are often based on the strategy to convert organic and ammonia-nitrogen first to nitrate (nitrification; requires oxic conditions) and later transform nitrate to molecular nitrogen (denitrification; requires anoxic conditions). N_2_O can be formed via intermediary products in both nitrification and denitrification.^1^ Accordingly, N_2_O emissions from this treatment step often exhibit short-term variability over hourly to diel time frames, in correspondence with nitrogen loads and the shifts between aerobic and anaerobic conditions in the treatment processes.^26^ Recently, the importance of N_2_O emissions from nitrogen treatment steps in WWTPs was highlighted, and the assessment methods were challenged, calling for more diversified approaches and more measurements.^27^

The results of large N_2_O emissions from the anaerobic digestion sludge deposits, assumed to be negligible in the IPCC emission assessment methodology, are not surprising from a mechanistic perspective. Substantial N_2_O emissions are well-known from composting of sludge^17,18^ and while sludge from AD may not release N_2_O in the AD chamber, the sludge surface layers will, during outdoor storage, be subjected to intrusion of molecular oxygen (O_2_), forming redox gradients which whish should resemble compost piles, and where favorable conditions for N_2_O formation are very likely.

As can be seen in Table 2, there are very few studies on N_2_O emissions from wastewater AD sludge (with very few facilities included in each study). All of these studies focus on studying AD sludge in the lab environment using point measurements. This study uses clouds of in situ samples around full-scale sludge piles under real-world outside conditions (wind, precipitation, temperature, sunlight, and other related everyday WWTP activities at the sludge piles) for six facilities.

All of the studies in Table 2 observed significant N_2_O release, and in some cases, in amounts that dominated over CH_4_ release in terms of CO_2_-equivalent contributions. Accordingly, our findings are consistent with both the mechanistic understanding of N_2_O formation during the storage of nitrogen-rich organic sludge and recent literature evidence from multiple independent studies.

The sludge pile emissions are disconnected from variability in N load or from specific treatment cycles (in contrast to N_2_O emissions from the N removal steps). Hence, short-term temporal variability of digestate emissions is likely to be irregular and may depend on temperature, variability in O_2_ (which may depend on sludge pile exposure to wind and rain), pH, ammonium, and the C/N ratio.^16–18,27^ In addition, over a monthly to yearly scale, the most important factor could also be sludge storage time, with most emissions coming from younger piles.^14,16^ At present, in the absence of detailed in situ studies of temporal dynamics regarding sludge N_2_O emissions, the flux regulation can only be hypothesized based on results from incubation studies and from N_2_O emissions in other environments (e.g., nitrogen removal treatments and compost piles).^16–18,27^ To learn more about flux regulation, one option is conducting detailed studies of flux dynamics from full-scale sludge piles exposed to experimental treatments regarding e.g., wind, moisture, temperature (external and internal), sludge age, and sludge types and pretreatments. The drone method, as presented, would allow such studies of flux regulation and also bring the capacity to test different ways to mitigate sludge emissions.

Our integrated measurements of the total sludge storage at the WWTPs include sludge at all storage times, thereby providing a realistic mean over time in terms of sludge age (0–6 months) and also covering a wide range of weather conditions (−1 to +27 °C and months March to August) considering the combined measurements at all WWTPs. None of the WWTPs in this study, or any WWTPs that we are aware of, measure N_2_O emissions from sludge piles. The WWTPs do, however, measure CH_4_ regularly but not the emissions from full-scale sludge piles, as few methods are available that allow direct measurements of full-scale sludge pile emissions, with the possibility to separate different treatment steps and different sludge piles from each other. Using the drone method, or a similar method covering full-scale sludge piles, it would be interesting to study in further detail how the CH_4_ and N_2_O emissions vary with seasons and digestion temperature (mesophilic versus thermophilic).

Results are presented in greater detail in the Supporting Information, including the scaling of CH_4_ and N_2_O flux with person equivalents (Figure S1), measurements of individual process steps (Table S1), a comparison of sludge GHG CH_4_ and N_2_O (Table S2), and manual air samples at sludge piles confirming the high N_2_O levels found (Table S3). The Supporting Information also includes a method discussion.

The large underestimates revealed in this study illustrate that current knowledge of GHG emissions from WWTPs with AD is inaccurate. In turn, this means that comparisons among WWTPs with different treatment and sludge management strategies may be biased. Until this situation is resolved, and more accurate and comprehensive emission assessments are available, telling where, when, and how much is emitted, it is challenging to develop effective GHG flux mitigation strategies. Hence, the development and implementation of accurate measurement approaches should have high priority.

Our results call for a reevaluation of WWTP GHG emissions. Further, flux mitigation development is necessary to decrease present emissions and prevent increased emissions due to the ongoing global WWTP expansion. There is a rich body of literature on GHG mitigation strategies regarding nitrogen treatments^34^ and on how to reduce energy and fossil fuel needs.^21^ Some alternatives have been tested successfully to mitigate emissions from sludge, but trade-offs among different approaches call for further development and optimization. For example, thermogenic digestion seems to reduce CH_4_ emissions from sludge but may increase the corresponding N_2_O emissions.^16^ Addition of ammonia or urea has been shown to reduce both N_2_O and CH_4_ emissions but adds a cost and risk of increased unwanted N emissions beyond the sludge storage period. Mitigation approaches tested for aerobic sludge composting can also be of interest, such as addition of biochar and manganese ore.^35^

Notably, there was great interest in mitigating GHG emissions at all the WWTPs visited, and the results triggered local mitigation planning and preparations for associated investments. This indicates that GHG flux mitigation is already a high priority for many municipal WWTPs and that the capacity to develop and implement mitigation approaches is high. The primary limitations to effective mitigation may be the scarcity of comprehensive and cost-effective in situ observation approaches that can identify emissions, justify the necessary local investments, and evaluate mitigation success.^14,24^ The emerging iUAS, along with the established tracer-gas methodologies, can be important tools in resolving this situation. Also, the discovery of large N_2_O emissions from full-scale sludge piles could motivate voluntary commitment programs, and the iUAS method itself could be used to prioritize actions to lower emissions and follow up on their efficiency.

## Supplementary Material



## Notes

Additional Figures S1–S5; additional Tables S1–S4; treatment steps used at the WWTPs in this study; method overview for measuring WWTP GHG emissions; drone method discussion (PDF)

## References

[1] United Nations Human Settlements Programme. Progress on Wastewater Treatment Global Status and Acceleration Needs for SDG Indicator 6.3.1; World Health Organization: Geneva, 2021.

[2] Jones E. R., Van Vliet M. T. H., Qadir M., Bierkens M. F. P. (2021). Country-Level
and Gridded Estimates of Wastewater Production, Collection, Treatment
and Reuse. Earth Syst. Sci. Data.

[3] De
Haas D., Andrews J. (2022). Nitrous Oxide Emissions from Wastewater Treatment -
Revisiting the IPCC 2019 Refinement Guidelines. Environ. Challenges.

[4] Sieranen M., Hilander H., Haimi H., Larsson T., Kuokkanen A., Mikola A. (2024). Seasonality of Nitrous Oxide Emissions at Six Full-Scale
Wastewater Treatment Plants. Water Sci. Technol..

[5] Tumendelger A., Alshboul Z., Lorke A. (2019). Methane and Nitrous Oxide Emission
from Different Treatment Units of Municipal Wastewater Treatment Plants
in Southwest Germany. PLoS One.

[6] Michael
Fredenslund A., Gudmundsson E., Maria Falk J., Scheutz C. (2023). The Danish National Effort to Minimise Methane Emissions
from Biogas Plants. Waste Manage..

[7] IPCC Guidelines for National Greenhouse Gas Inventories, Institute for Global Environmental Strategies: Hayama, Japan, 2006.

[8] Buendia, E. C. ; Tanabe, K. ; Kranjc, A. ; Jamsranjav, B. ; Fukuda, M. ; Ngarize, S. ; Osako, A. ; Pyrozhenko, Y. ; Shermanau, P. ; Federici, S. 2019 IPCC Refinement to the 2006 IPCC Guidelines for National Greenhouse Gas Inventories; Institute for Global Environmental Strategies: Hayama, Japan, 2019.

[9] Saunois M., Stavert A. R., Poulter B., Bousquet P., Canadell J. G., Jackson R. B., Raymond P. A., Dlugokencky E. J., Houweling S., Patra P. K., Ciais P., Arora V. K., Bastviken D., Bergamaschi P., Blake D. R., Brailsford G., Bruhwiler L., Carlson K. M., Carrol M., Castaldi S., Chandra N., Crevoisier C., Crill P. M., Covey K., Curry C. L., Etiope G., Frankenberg C., Gedney N., Hegglin M. I., Höglund-Isaksson L., Hugelius G., Ishizawa M., Ito A., Janssens-Maenhout G., Jensen K. M., Joos F., Kleinen T., Krummel P. B., Langenfelds R. L., Laruelle G. G., Liu L., Machida T., Maksyutov S., McDonald K. C., McNorton J., Miller P. A., Melton J. R., Morino I., Müller J., Murguia-Flores F., Naik V., Niwa Y., Noce S., O’Doherty S., Parker R. J., Peng C., Peng S., Peters G. P., Prigent C., Prinn R., Ramonet M., Regnier P., Riley W. J., Rosentreter J. A., Segers A., Simpson I. J., Shi H., Smith S. J., Steele L. P., Thornton B. F., Tian H., Tohjima Y., Tubiello F. N., Tsuruta A., Viovy N., Voulgarakis A., Weber T. S., Van Weele M., Van Der Werf G. R., Weiss R. F., Worthy D., Wunch D., Yin Y., Yoshida Y., Zhang W., Zhang Z., Zhao Y., Zheng B., Zhu Q., Zhu Q., Zhuang Q. (2020). The Global
Methane Budget 2000–2017. Earth Syst.
Sci. Data.

[10] Bassin, J. P. ; Castro, F. D. ; Valério, R. R. ; Santiago, E. P. ; Lemos, F. R. ; Bassin, I. D. The Impact of Wastewater Treatment Plants on Global Climate Change. In Water Conservation in the Era of Global Climate Change; Elsevier, 2021, pp. 367–410 10.1016/B978-0-12-820200-5.00001-4.

[11] Tian H., Pan N., Thompson R. L., Canadell J. G., Suntharalingam P., Regnier P., Davidson E. A., Prather M., Ciais P., Muntean M., Pan S., Winiwarter W., Zaehle S., Zhou F., Jackson R. B., Bange H. W., Berthet S., Bian Z., Bianchi D., Bouwman A. F., Buitenhuis E. T., Dutton G., Hu M., Ito A., Jain A. K., Jeltsch-Thömmes A., Joos F., Kou-Giesbrecht S., Krummel P. B., Lan X., Landolfi A., Lauerwald R., Li Y., Lu C., Maavara T., Manizza M., Millet D. B., Mühle J., Patra P. K., Peters G. P., Qin X., Raymond P., Resplandy L., Rosentreter J. A., Shi H., Sun Q., Tonina D., Tubiello F. N., Van Der Werf G. R., Vuichard N., Wang J., Wells K. C., Western L. M., Wilson C., Yang J., Yao Y., You Y., Zhu Q. (2024). Global Nitrous Oxide Budget (1980–2020). Earth Syst. Sci. Data.

[12] Paredes M. G., Güereca L. P., Molina L. T., Noyola A. (2015). Methane Emissions from
Stabilization Ponds for Municipal Wastewater Treatment in Mexico. J. Integr. Environ. Sci..

[13] Moore D. P., Li N. P., Wendt L. P., Castañeda S. R., Falinski M. M., Zhu J.-J., Song C., Ren Z. J., Zondlo M. A. (2023). Underestimation of Sector-Wide Methane
Emissions from
United States Wastewater Treatment. Environ.
Sci. Technol..

[14] Gålfalk M., Påledal S. N., Sehlén R., Bastviken D. (2022). Ground-Based
Remote Sensing of CH4 and N2O Fluxes from a Wastewater Treatment Plant
and Nearby Biogas Production with Discoveries of Unexpected Sources. Environ. Res..

[15] Ahn J. H., Kim S., Park H., Rahm B., Pagilla K., Chandran K. (2010). N _2_ O Emissions from Activated Sludge Processes, 2008–2009: Results
of a National Monitoring Survey in the United States. Environ. Sci. Technol..

[16] Willén A., Rodhe L., Pell M., Jönsson H. (2016). Nitrous Oxide
and Methane Emissions during Storage of Dewatered Digested Sewage
Sludge. J. Environ. Manage..

[17] Nordahl S. L., Preble C. V., Kirchstetter T. W., Scown C. D. (2023). Greenhouse Gas and
Air Pollutant Emissions from Composting. Environ.
Sci. Technol..

[18] Czepiel P., Douglas E., Harriss R., Crill P. (1996). Measurements of N_2_ O from Composted Organic Wastes. Environ.
Sci. Technol..

[19] Gålfalk M., Påledal S. N., Yngvesson J., Bastviken D. (2024). Measurements
of Methane Emissions from a Biofertilizer Storage Tank Using Ground-Based
Hyperspectral Imaging and Flux Chambers. Environ.
Sci. Technol..

[20] Gålfalk M., Nilsson Påledal S., Bastviken D. (2021). Sensitive Drone Mapping of Methane
Emissions without the Need for Supplementary Ground-Based Measurements. ACS Earth Space Chem..

[21] Mayer F., Bhandari R., Gäth S. (2019). Critical Review
on Life Cycle Assessment
of Conventional and Innovative Waste-to-Energy Technologies. Sci. Total Environ..

[22] Gao H., Scherson Y. D., Wells G. F. (2014). Towards
Energy Neutral Wastewater
Treatment: Methodology and State of the Art. Environ. Sci.: Processes Impacts.

[23] Shaw J. T., Shah A., Yong H., Allen G. (2021). Methods for Quantifying
Methane Emissions Using Unmanned Aerial Vehicles: A Review. Philos. Trans. R. Soc., A.

[24] Bastviken D., Wilk J., Duc N. T., Gålfalk M., Karlson M., Neset T.-S., Opach T., Enrich-Prast A., Sundgren I. (2022). Critical Method Needs in Measuring
Greenhouse Gas Fluxes. Environ. Res. Lett..

[25] Intergovernmental Panel On Climate Change (Ipcc) Climate Change 2021 – The Physical Science Basis: Working Group I Contribution to the Sixth Assessment Report of the Intergovernmental Panel on Climate Change; Cambridge University Press, 2023. 10.1017/9781009157896.

[26] Daelman M. R. J., Van Voorthuizen E. M., Van Dongen U. G. J. M., Volcke E. I. P., Van Loosdrecht M. C.
M. (2015). Seasonal and Diurnal
Variability of N 2 O Emissions from a Full-Scale Municipal Wastewater
Treatment Plant. Sci. Total Environ..

[27] Song C., Zhu J.-J., Willis J. L., Moore D. P., Zondlo M. A., Ren Z. J. (2024). Oversimplification
and Misestimation of Nitrous Oxide
Emissions from Wastewater Treatment Plants. Nat. Sustain..

[28] Colón J., Cadena E., Pognani M., Barrena R., Sánchez A., Font X., Artola A. (2012). Determination of the
Energy and Environmental
Burdens Associated with the Biological Treatment of Source-Separated
Municipal Solid Wastes. Energy Environ. Sci..

[29] Maulini-Duran C., Artola A., Font X., Sánchez A. (2013). A Systematic
Study of the Gaseous Emissions from Biosolids Composting: Raw Sludge
versus Anaerobically Digested Sludge. Bioresour.
Technol..

[30] Beylot A., Vaxelaire S., Zdanevitch I., Auvinet N., Villeneuve J. (2015). Life Cycle
Assessment of Mechanical Biological Pre-Treatment of Municipal Solid
Waste: A Case Study. Waste Manage..

[31] Zeng Y., De Guardia A., Dabert P. (2016). Improving Composting as a Post-Treatment
of Anaerobic Digestate. Bioresour. Technol..

[32] Li Y., Luo W., Lu J., Zhang X., Li S., Wu Y., Li G. (2018). Effects of
Digestion Time in Anaerobic Digestion on Subsequent Digestate
Composting. Bioresour. Technol..

[33] Preble C. V., Chen S. S., Hotchi T., Sohn M. D., Maddalena R. L., Russell M. L., Brown N. J., Scown C. D., Kirchstetter T. W. (2020). Air Pollutant
Emission Rates for Dry Anaerobic Digestion and Composting of Organic
Municipal Solid Waste. Environ. Sci. Technol..

[34] Duan H., Van Den Akker B., Thwaites B. J., Peng L., Herman C., Pan Y., Ni B.-J., Watt S., Yuan Z., Ye L. (2020). Mitigating
Nitrous Oxide Emissions at a Full-Scale Wastewater Treatment Plant. Water Res..

[35] Zhou S., Li Y., Jia P., Wang X., Kong F., Jiang Z. (2022). The Co-Addition
of Biochar and Manganese Ore Promotes Nitrous Oxide Reduction but
Favors Methane Emission in Sewage Sludge Composting. J. Cleaner Prod..

