# Supplementary material for: In Situ Observations Reveal Underestimated Greenhouse Gas Emissions from Wastewater Treatment with Anaerobic Digestion – Sludge Was a Major Source for Both CH4 and N2O
Source: Environ Sci Technol. 2025 Aug 22;59(34):18146–55. doi: 10.1021/acs.est.5c04780 (PMC12409893; doi:10.1021/acs.est.5c04780)
Supplement: Supplementary file 1 [file es5c04780_si_001.pdf]

Supporting information

Title: In situ observations reveal underestimated greenhouse gas emissions from wastewater treatment with anaerobic digestion – sludge was a major source for both CH_4 and N_2O

Authors: Magnus **Gålfalk**[a,*], David **Bastviken**[a]
(family names written in bold)

[a]Department of Thematic Studies – Environmental Change, Linköping University, 581 83 Linköping, Sweden.
e-mails: magnus.galfalk@liu.se, david.bastviken@liu.se

Number of supporting figures: 5
Number of supporting tables: 4
Additional text: treatment steps used at the WWTPs in this study
Additional text: method overview for measuring WWTP GHG emissions
Additional text: drone method discussion
Pages: 17

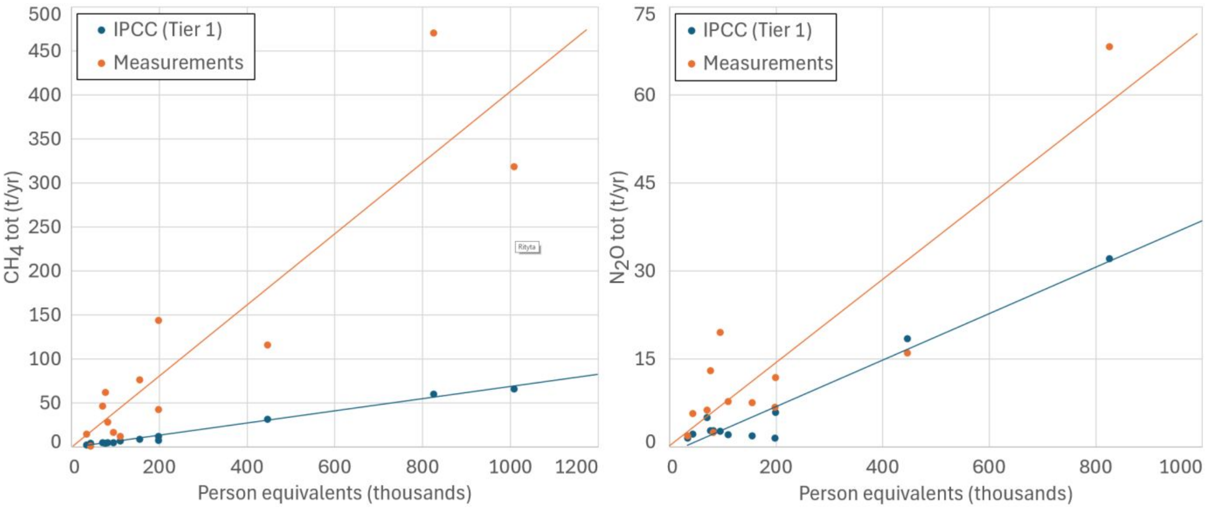

Figure S1. Total measured CH_4 (left panel) and N_2O (right panel) WWTP emissions compared to IPCC estimates for WWTP loads up to 1 000 000 person equivalents. The solid lines are regression lines for the measurement and IPCC data points, respectively. Please note that these graphs are not intended as predictive models but rather to compare the in-situ observations with IPCC Tier 1 estimates by person equivalents loads.

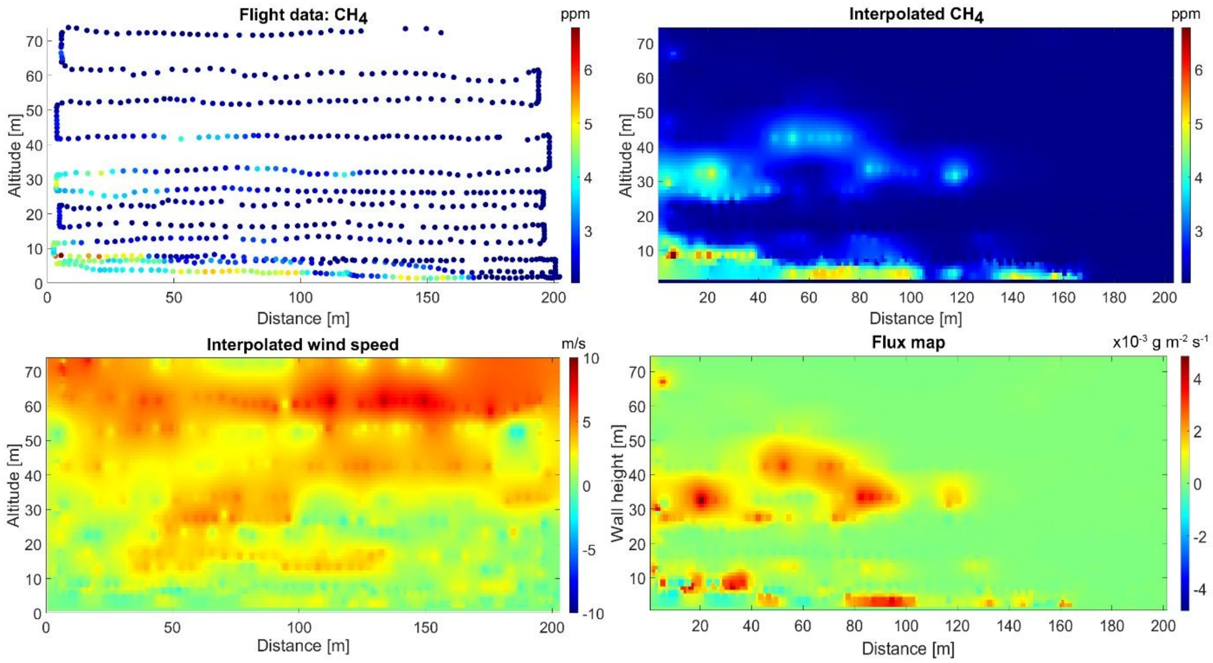

Figure S2. Example of a flux wall calculation (rightmost wall in Figure 1 for CH_4). The panels show the point cloud of CH_4 concentrations, interpolated and hole-filled CH_4 map, the corresponding interpolated and hole-filled wind speed map, and the resulting CH_4 flux map that is integrated to calculate the total CH_4 flux through this wall.

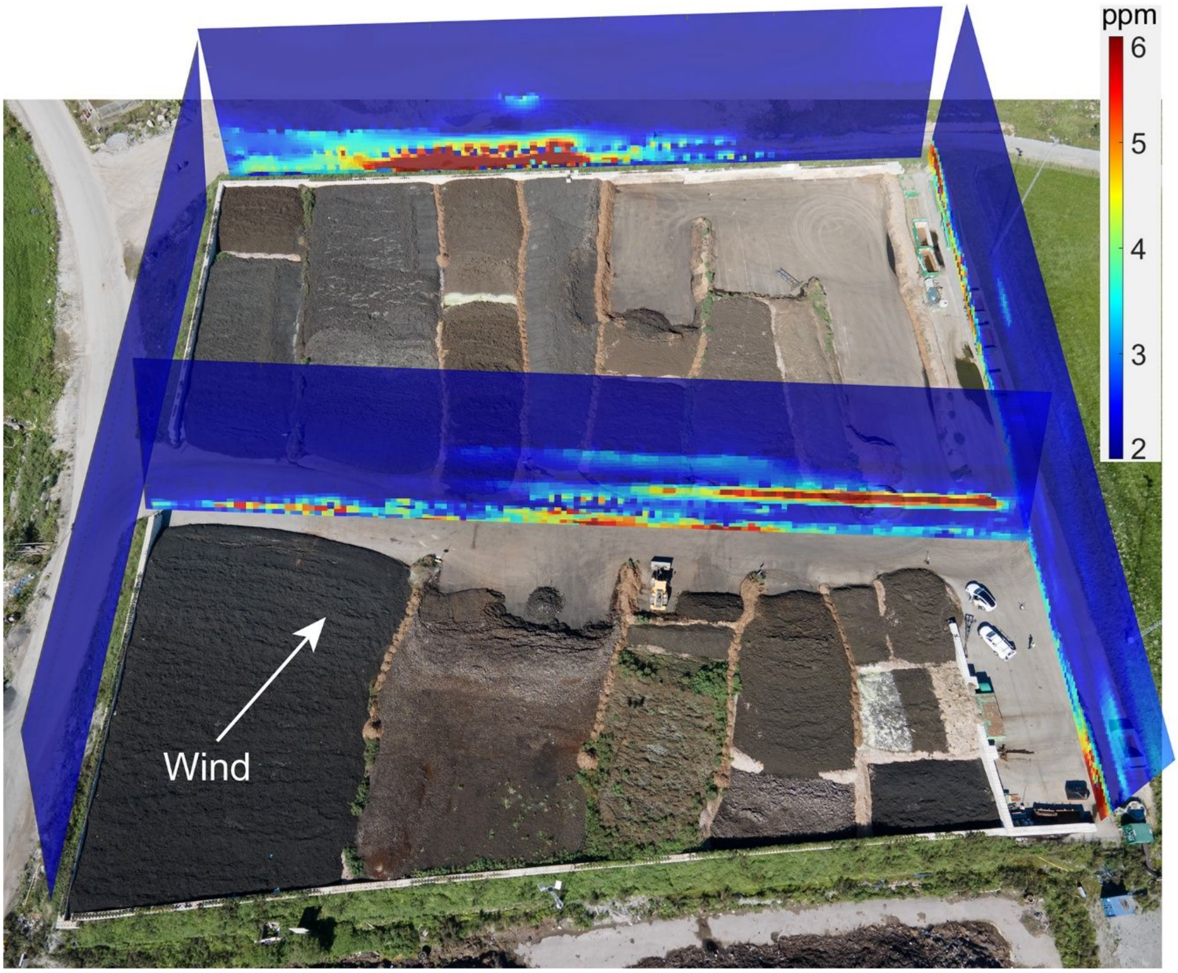

Figure S3. Vertical iUAS CH_4 concentration flight walls at a large sludge storage site with piles of different ages.

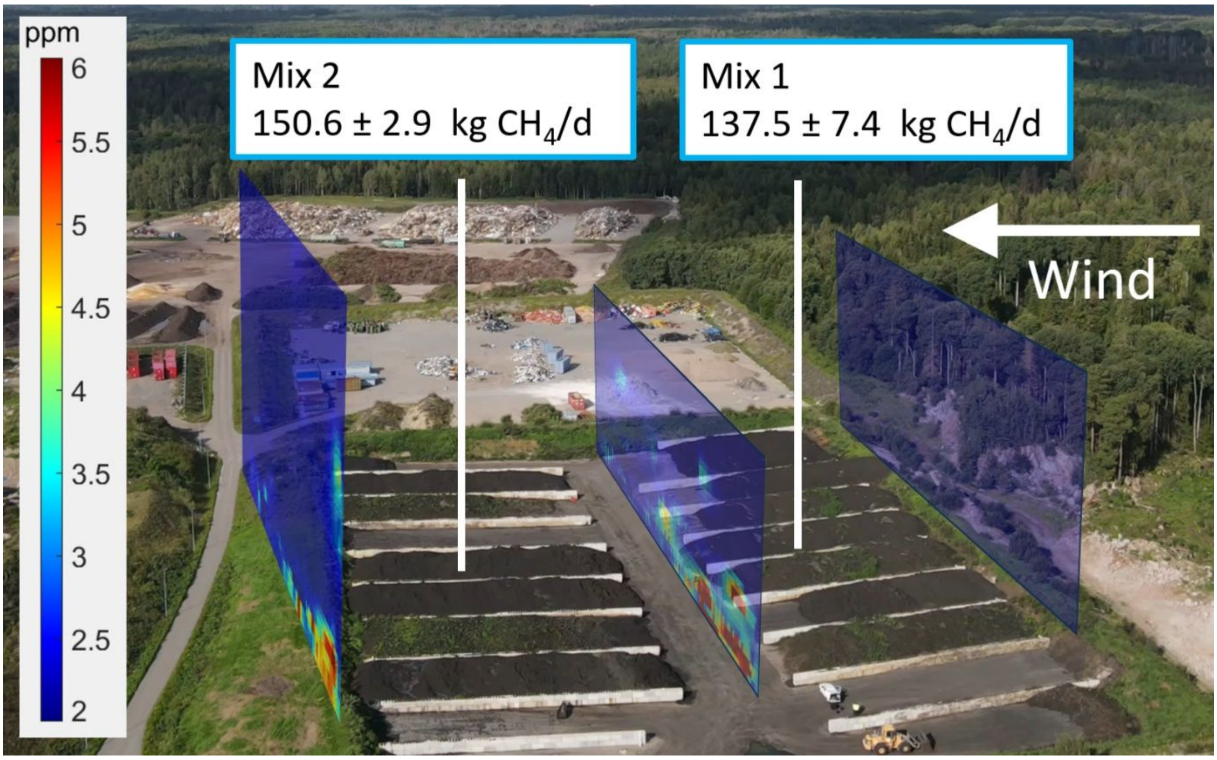

Figure S4. Vertical iUAS CH_4 concentration flight walls at a large sludge storage site with piles of different ages (a new pile each month).

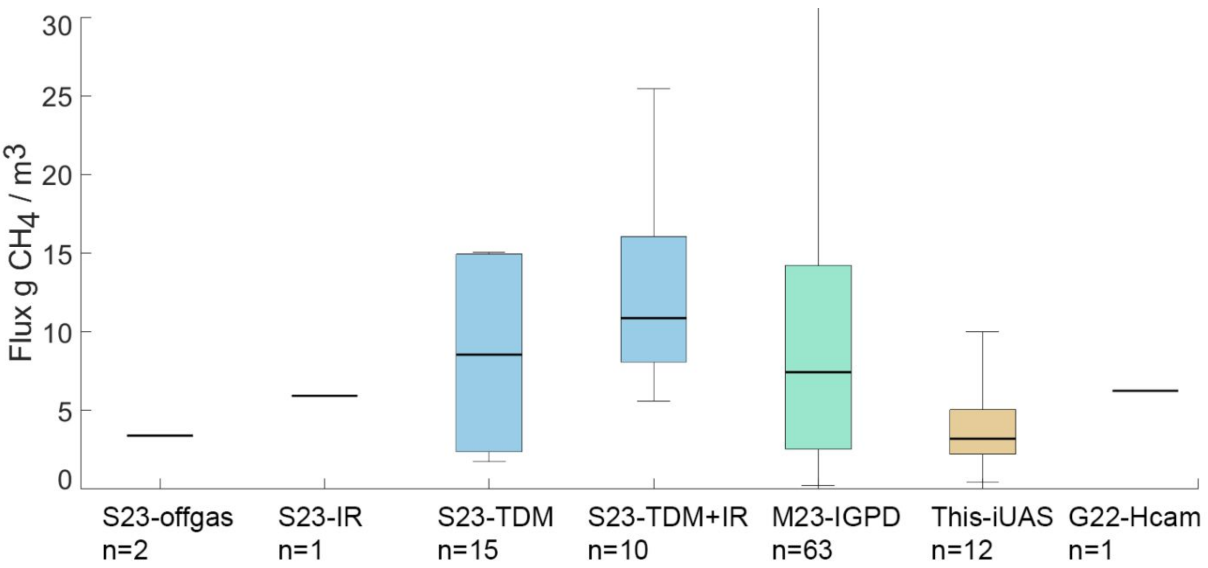

Figure S5. A comparison of WWTP CH_4 emissions in this and previous studies in literature. Each bar represents different methods (TDM: Tracer gas dispersion method, IGPD: Integrated Gaussian Plume Dispersion, Hcam: Hyperspectral camera) and studies (S23: Song et al. 2023, M23: Moore et al. 2023, G22: Gålfalk et al. 2022) with fluxes that have been normalized to gram CH_4 per m^3 of wastewater treated. Below each study and method, the number of WWTPs are given. For S23 we have included all WWTPs having anaerobic digestion (AD), and M23 consists of a mix of WWTPs with and without AD. The iUAS measurements in this study agree with the CH_4 emissions of the other studies (perhaps on the lower end as this study does not include all the treatment processed at each plant, instead being conservative by targeting the highest emitting processes; see Table 1).

Table S1. Measurements of targeted CH_4 and N_2O sources using iUAS systems 1 and 2. Flux values represent means ± 1 SD.

WWTP	Target	CH_4 emission *kg/d*	N_2O emission *kg/d*	UAS system	Date
1	Reject water tank (no treatment)	-	15.5 ± 1.2	1	2022-07-12
	Sludge deposit (0-1.5 mo)	10.1 ± 0.2	-	1	2022-07-12
2	Reject water tank (ARP)	-	8.5 ± 0.9	1	2022-07-13
	Biological treat.	-	9.9 ± 0.6	1	2022-07-13
	Sludge deposit (0-1 wk) with bark filter	34.1 ± 4.0	-	1	2022-07-13
3	Biological treatment (nitrification)	-	6.4 ± 0.9	1	2022-07-15
	Sludge deposits (0-1 mo)	129 ± 20	-	1	2022-07-15
4	Biological treatment (activated sludge)	-	11.8 ± 1.7	1	2022-07-26
	Biological treatment (biobeds, nitrification)	-	9.3 ± 1.8	1	2022-07-26
	Sludge deposit (0-6 mo)	269 ± 7	-	1	2022-07-26
	Open sludge tank	50 ± 6	-	1	2022-07-26
5	Sludge deposit (0-3 mo)	62.7 ± 1.6	-	1	2022-07-27
	Sand filter	0 ± 5	0.41 ± 0.08	1	2022-07-27
	Biological treatment (activated sludge)	15 ± 11	1.35 ± 0.38	1	2022-07-27
6	Biological treatment 1 (activated sludge)	40.1 ± 2.6	34.5 ± 1.7	1	2022-07-28
	Biological treatment 2 (activated sludge)	5.8 ± 0.9	19.2 ± 1.7	1	2022-07-28
7	Sludge deposit (0-6 mo)	230 ± 4	-	1	2022-03-23
	Sludge deposit (0.5-3.5 mo)	118.1 ± 5.6	8.08 ± 0.89	2	2023-06-16
	Reject water tank (SHARON) still	-	1.2 ± 1.5	1	2022-03-24
	Reject water tank (SHARON) aeration	-	20.3 ± 1.4	1	2022-03-24
8	Sludge deposit (0-1 mo)	210 ± 12	-	1	2022-06-30
	Reject water tank (DEMON)	-	1.03 ± 0.20	1	2022-06-30
	Biological treatment (activated sludge)	-	1.70 ± 0.20	1	2022-06-30
9	Reject water tanks (ANAMMOX)	34.7 ± 12.6	85.8 ± 5.6	2	2023-08-23
	Post-nitrification	8.68 ± 0.84	3.14 ± 0.32	2	2023-08-24
	Chimney at post-nitrification	6.63 ± 0.81	10.91 ± 0.73	2	2023-08-24
	Sedimentation tanks	283.5 ± 4.3	3.61 ± 0.59	2	2023-08-24
	Sludge deposit (mix. 1)	73.6 ± 5.4	6.58 ± 1.16	2	2023-08-24
	Sludge deposit (mix. 2)	788.9 ± 20.3	48.6 ± 2.3	2	2023-08-24
	Sludge deposit (mix. 3) (incl. compost mat.)	94.7 ± 6.3	28.4 ± 2.5	2	2023-08-24
	Sludge deposit (total) (0-6 mo)	957 ± 22	83.6 ± 3.6	2	2023-08-24
10	Biological treatment (sequencing batch reactor)	27.97 ± 1.93	15.54 ± 2.31	2	2023-06-26
	Biological treatment (activated sludge)	2.67 ± 2.14	8.15 ± 0.80	2	2023-06-26
	Sludge deposit (0-1 w)	140.4 ± 11.3	-0.7 ± 2.4	2	2023-06-26
11	Sludge deposit (0-1 mo)	25.8 ± 2.4	2.61 ± 0.16	2	2023-08-28
	Sludge deposit (1-2 mo)	11.6 ± 1.5	1.82 ± 0.25	2	2023-08-28
	Sludge deposit (2-6 mo)	4.1 ± 1.3	0.87 ± 0.18	2	2023-08-28

	Sludge deposit (0-1 mo)	8.0 ± 0.8	0.10 ± 0.07	2	2024-04-04
	Sludge deposit (1-2 mo)	7.2 ± 1.2	0.09 ± 0.15	2	2024-04-04
	Sludge deposit (2-6 mo)	2.5 ± 1.0	0.18 ± 0.22	2	2024-04-04
	Biological treatment (biobeds, nitrification)	14.7 ± 1.9	9.58 ± 0.28	2	2024-04-04
12	Biological treatment 1 (activated sludge)	3.48 ± 0.86	0	2	2023-08-30
	Biological treatment 2 (activated sludge)	103.1 ± 14.1	14.13 ± 1.98	2	2023-08-30
	Sludge deposit (mix. 1)	137.5 ± 7.4	5.18 ± 0.42	2	2023-08-31
	Sludge deposit (mix. 2)	150.6 ± 2.9	13.31 ± 0.29	2	2023-08-31
	Sludge deposit (total) (0-6 mo)	288.1 ± 8.0	18.49 ± 0.52	2	2023-08-31
13	Sludge deposit (0-6 mo)	874.1 ± 31.8	71.31 ± 2.20	2	2023-08-29

Table S2. A comparison of sludge GHG fluxes both as CH_4, N_2O, and converted to CO_2 equivalents over a 100 yr timescale. Flux values represent means ± 1 SD.

WWTP	Age *mo*	CH_4 *kg CH_4/tonDM,d*	N_2O *kg N_2O/tonDM,d*	CH_4 (CO_2eq) *kg CO_2/tonDM,d*	N_2O (CO_2eq) *kg CO_2/tonDM,d*	N_2O/CH_4 *CO_2eq*
7	0.5-3.5	0.230 ± 0.011	0.016 ± 0.002	6.45 ± 0.31	4.70 ± 0.52	0.73
9	0-6	0.126 ± 0.003	0.011 ± 0.001	3.53 ± 0.09	3.29 ± 0.15	0.93
11	0-1	0.550 ± 0.052	0.056 ± 0.004	15.40 ± 1.44	16.58 ± 1.02	1.08
	1-2	0.247 ± 0.032	0.039 ± 0.006	6.92 ± 0.90	11.56 ± 1.59	1.67
	2-6	0.022 ± 0.007	0.005 ± 0.001	0.61 ± 0.20	1.38 ± 0.29	2.26
12	0-6	0.175 ± 0.005	0.011 ± 0.001	4.89 ± 0.14	3.34 ± 0.10	0.68
13	0-6	0.096 ± 0.004	0.008 ± 0.001	2.68 ± 0.10	2.33 ± 0.08	0.87

Table S3. Manual air samples by syringe over sludge deposits at WWTPs 7 and 8 at a height of 0.5 m from an earlier pilot study confirming elevated mixing ratios of both gases compared to background levels, in turn showing emissions. Note that individual point samples are not representative for the whole sludge pile nor adequate for emission calculations and just confirm emissions qualitatively by independent sampling. Excess concentrations are given for CH_4 and N_2O relative to the ambient concentration far from the sludge piles. The median excess N_2O/CH_4 ratio is 0.061 (indicating N_2O emissions 6.1% of the CH_4 emissions by concentration or equivalently 16.8% by mass).

WWTP	Sludge age	Excess CH_4 *ppm*	Excess N_2O *ppm*	Excess N_2O/CH_4	Date
7	< 24 d (p1)	6.48	0.11	0.017	2018-10-24
	< 24 d (p2)	1.94	0.08	0.041	
	< 24 d (p3)	5.54	0.15	0.027	
	< 24 d (p4)	14.24	0.18	0.013	
7	43-73 d (p1)	2.08	0.10	0.048	2018-11-12
	43-73 d (p2)	3.50	0.14	0.040	
	43-73 d (p3)	9.48	0.41	0.043	
7	20-48 d	4.07	0.13	0.032	2019-03-20
	49-69 d (p1)	0.31	0.13	0.419	
	49-69 d (p2)	0.42	0.13	0.310	
7	0-17 d (p1)	5.49	0.35	0.064	2019-06-17
	0-17 d (p2)	26.20	2.03	0.078	
	18-48 d (p1)	15.17	3.07	0.202	
	18-48 d (p2)	2.09	0.17	0.081	
7	10-40 d	15.55	0.33	0.021	2019-09-10
	41-70 d (p1)	5.17	1.47	0.284	
	41-70 d (p2)	3.75	1.17	0.312	
	41-70 d (p3)	7.60	2.66	0.350	
8	0-28 d	1.84	0.05	0.027	2019-06-28
	29-59 d	0.74	0.06	0.081	
	60-90 d	2.16	0.21	0.097	
8	0-26 d	61.0	0.13	0.002	2019-08-26
	27-57 d	1.66	0.18	0.108	
	58-87 d	0.52	0.03	0.058	2019-08-26

Table S4. An overview of the treatment steps at the WWTPs (more details on step 3B are given in Table S1). The symbol "X" indicates that a WWTP uses that treatment step while "/" indicates that sludge is produced but stored less than one week or not at all on-site. WWTP13 is a large facility for anaerobically digested sludge storage with deliveries from other WWTPs.

Treatment process	WWTP												
	1	2	3	4	5	6	7	8	9	10	11	12	13
1. Mechanical grit removal	X	X	X	X	X	X	X	X	X	X	X	X	
2. Chemical flocculation	X	X	X	X	X	X	X	X	X	X	X	X	
3A. Biological treatment - organic matter and nitrogen removal	X	X	X	X	X	X	X	X	X	X	X	X	
3B. Biological treatment – reject water tank		X	X	X	X		X	X	X	X	X		
4. Harvesting of particles	X	X	X	X	X	X	X	X	X	X	X	X	
5. Polishing of water			X	X	X	X	X	X	X				
6. Sludge collection and anaerobic digestion	X	X	X	X	X	X	X	X	X	X	X	X	
7. Sludge storage of anaerobically digested sludge	X	/	X	X	X	/	X	X	X	/	X	X	X

Treatment steps used at the different WWTPs

The studied WWTPs shared a general design and treatment processes which are common in Swedish WWTPS. A general description is provided in the materials and methods section, with further, more detailed descriptions below and table S4 showing which processes belong to each WWTP.

Table S1 gives the emissions for all measured treatment steps and WWTPs included in the study. The focus has been on measuring sludge storage piles (from anaerobic digestion; storage from 0-6 mo), reject water tanks (different techniques given below), and biological treatment steps. The reason for this focus is that these treatment steps had been identified as the highest emitters of CH_4 in our pre-study of all treatment steps at a WWTP (Gålfalk et al. 2022).

In Sweden anaerobically digested sludge is stored 6-12 months (depending on WWTP) in large piles (often one for each month) in an open space outside for mesophilic sludge and thereafter spread on farmland. In the rarer case of thermophile sludge (higher temperatures; not included in this study) the sludge would not have to be stored (but emissions would instead occur on farmland). A typical sludge pile in our study is 35 x 15 x 2.5 meters, but they can also be much smaller (down to about 5 meters) or much larger (e.g. merged piles with area sizes of 150 x 100 meters).

Pre-sedimentation basins are used to settle formed flocks, and sedimented material is then pumped to digestion chambers for anaerobic digestion. After this remaining sludge is stored in a buffer tank and dewatered using a screw press and finally placed in the large outside storage space where we measured emissions with the drone-method.

The reject water tanks used different treatment methods but had the purpose of nitrogen (ammonia) removal, using water from the screw press prior to this step. One version uses the SHARON (Stable High rate Ammonia Removal Over Nitrite) process involving an open basin with a continuous flow of water and alternating cycles of nitrification and denitrification. Another version is ARP (Activated Return sludge Process) which also uses nitrification and denitrification, in combination with hydrolysis and bio adsorption. One of the WWTPs used the DEMON (DE-amMONification) reactor which is a continuous single stage process. The ANAMMOX (ANaerobic AmmoniuM OXidation) process is used in one of the WWTPs (nitrification and denitrification in one step).

Biological treatment was mostly done by the activated sludge method which consisted of long basins with aeration and sedimentation to remove organic material and nitrogen (nitrification / denitrification). A sequencing batch reactor works in a similar way but using only one basin.

Many WWTPs in Sweden participate in a voluntary commitment called EgMet to measure CH_4 emissionvs every 3rd year, with methods often being point-based such as sampling in ventilation systems (Avfall Sverige 2019). If sludge is included the methods are typically based on gas release from bottle-incubated samples in a lab, or attempted flux chamber measurements from a small part of a pile (not representative of the full-scale sludge pile). N_2O emissions are not measured regularly, and the biological treatment is typically in focus. We have motivated the usage of a drone-based method for this study below in a method overview.

Method overview for measuring WWTP GHG emissions

In the following method overview, the pros and cons of different methods for measuring GHG emissions at WWTPs will be discussed. The emission source areas in a WWTP are highly variable and range from point emissions via ventilation outlets (a few cm in diameter at well-defined locations) to hectare-scale areas where sludge piles are stored and from which fluxes are highly heterogeneous in space. Ideally, GHG emission measurement methods should accurately quantify this full range of

emissions accurately, with capacity to locate emission sources (i.e. having well-constrained flux footprint areas), and operate by similar principles for all flux types. The method hence needs to be adaptable to large differences in scale, sensitive enough to detect the lower distributed emissions of older sludge piles, and accurate enough in footprints to separate sludge pile fluxes from other sources in a complicated industrial area such as a WWTP. For in situ sampling there is also the difficulty of representative sampling and difficult accessibility of required sampling points (such as on top of the middle of a sludge pile 2.5 m heigh, or the middle of a tank with water 30 meters in diameter). Methods typically used to approach these challenges are listed below. For more details about them see Bastviken et al. (2022) and the references therein.

1. **Static and flow-through flux chambers**
   Method: This approach is b*ased on a static chamber covering a footprint for a suitable period of time collecting gas, or a flow-through chamber with a known in- and outflow of air with a sensitive gas sensor measuring gas concentrations continuously.*
   Cons: Emission estimates require a very large number of measurements to be representative of a large surface (such as a sludge pile) to avoid bias as their footprints are typically only a few dm^2. For solid surfaces other cons are the uncertainty in keeping the surface-chamber interface airtight and the possible influence on fluxes from the contact. Further, wind influences are removed by the chamber with often unknown consequences for the flux results. Use on water surfaces is more straightforward but for solid and porous target areas (such as sludge piles) it is difficult to evaluate what flux chamber data represent.
   Pros: It is a well-established method known to give sensitive measurements for water surfaces, The material cost can be reasonably low and the handling and data processing is comparatively straightforward.

2. **Flux at outlets of well-defined point sources**
   Method: This could involve measuring air speed and gas concentrations at a ventilation outlet from a room in a facility or covering an emissions source with a gas sampling bag.
   Cons: It is only efficient for known point sources and is sensitive to precision and accuracy of concentrations measurements and the air transport speed. Does not work for distributed sources.
   Pros: The method is simple and straight-forward.

3. **Incubation of samples**
   Method: It involves taking samples of materials of interest (e.g. sludge) to a laboratory where samples are confined in vessels and gas concentrations are followed over time.
   Cons: Does not work for area fluxes as only point samples are taken. Does not represent the fluxes obtained in real-world conditions with outside temperatures, rain, wind, and temperature structure and other gradients inside or on a full-scale sludge pile. Emissions are only representative of the controlled environment conditions in the lab. Static incubations are not representative of the dynamic treatment processes elsewhere in the WWTP.
   Pros: Suitable for studying the influence of environmental parameters on gas production, revealing cause-effect relationships and regulation.

4. **Micrometeorological approaches by point measurements in ambient air at fixed positions.**
   Method: This involves methods such as Eddy Covariance (EC) towers, with high frequency and high precision sampling of gas concentrations and air motion. A model is used for tracing sampled air back to a corresponding footprint. This method is commonly used for e.g. forest and wetland fluxes but seems rare in the WWTP context.
   Cons: Footprint size and location changes continuously depending on wind direction and speed. The equipment is difficult or impossible (for larger towers) to move and thus mostly suitable for long-term studies and homogeneous environments (the opposite to WWTPs). It

is a demanding method regarding hardware specifications and calculations and comes with a high cost.
Pros: Emitting sources are not disturbed. Suitable for automatic long-term studies of larger homogeneous areas.

5. **Open approaches based on column density, tracers, or inverse modelling**
Method: *This is a group of methods that involves open path spectrometers (emitting light at certain wavelengths, reflectors, and a detector) to measure GHG fluxes from mass balance (downwind-upwind of a facility). A tracer gas placed at a location of interest could also be used as a marker of the footprint. Such tracer methods have been used to assess whole WWTP facility emissions.*
Cons: Dependent on weather conditions for obtaining the desired footprint. It can be difficult to separate nearby sources in an industrial environment. Tracer gas emissions could help for well-known points sources, but source confusion could exist for multiple point sources, unknown sources, or inhomogeneous extended sources.
Pros: The target areas are not disturbed. Accurate for known point sources. Inverse modeling could be used for many gases and source areas. Some of these methods also work for extended sources.

6. **Near-ground optical approaches with potential to map GHG concentrations and flux**
Method: *Passive optical gas detection methods use a cold or hot background (e.g. heated by sunlight) while active methods emit their own infrared light. Specialized infrared cameras have been used (e.g. a hyperspectral camera or a camera containing two gas cells with/without the gas of interest) to measure GHGs. To quantify gas concentrations, distances to the background also need to be mapped. Air movement is needed to assess flux which I some cases can be made by separate anemometers. In other cases, air movements can be derived from following gas movements over time in the camera data.*
Cons: The passive method is weather dependent as sunlight is often needed to heat up the background. These methods are not as sensitive as in situ measurements, e.g. N_2O has complicated absorption bands that also overlap H_2O bands. The very high equipment costs currently make these methods less available. High know-how is required for data interpretation.
Pros: Gives great visualizations of the otherwise invisible GHG emissions, with high efficiency in finding new sources anywhere in the field of view. These methods do not disturb the target area or the everyday activities at a facility. Fluxes can be calculated in post-processing for sources discovered after the measurements as long as they are in the field of view. Some hyperspectral camera systems can measure and visualize air motion (using naturally occurring H_2O as a tracer) at the same time as greenhouse gases.

7. **Mass balance from in situ measurements in a point cloud using a moving platform**
Method: *This class includes drones (fixed and rotary wing), helicopters, and aircraft. It involves sensitive high-frequency sampling of gas concentrations and wind speed and wind direction.*
Cons: All these methods are currently expensive to use and a high level of knowledge is required to use them. It can be difficult to measure in situ wind from a moving and rotating platform (but it is possible from a drone). Vertical air motion is affected beneath a drone or helicopter so care has to be taken when designing the system and flight path.
Pros: Emitting sources are not disturbed (if not flying too close). With a rotary wing drone, fluxes from different sources and multiple gases can be separated, independent of wind direction, as flight paths and speeds are adapted to give the desired footprints and spatial resolution. Large areas can be covered, and emissions can be measured that are difficult to access with other methods in a complex industrial landscape.

8. **Satellites**
   Method: New satellites are available that measure gases such as CH_4 from space
   Cons: Measurements so far only work for very high emissions sources and would therefore not be suitable for WWTPs. The spatial footprint is also yet rather large (km^2 scales). Air movement is required for flux estimation and is rarely available at corresponding scales and often rely on models.
   Pros: Large area coverage in consistent ways. Multiple ongoing initiatives aim to improve satellite GHG measurement capacity.

Given the suitability of a drone-based method for mapping GHG emissions at WWTPs we used this method for our study. The drone-based method stands out as particularly suitable for:

a) spatially flexible assessments of total net fluxes from 0.1 to 10 ha areas (including facilities or parts of facilities) with mobile equipment (same equipment at all sites reduces equipment bias),
b) having a well-defined measurement footprint,
c) enabling similar methodology regardless of land cover or activity on target area, and flux types (covers both point and distributed fluxes), and
d) avoiding measurements where physical disturbance of the target surface area can lead to large biases,
e) is sensitive and adaptable to wind conditions and capable of covering several GHGs simultaneously,
f) verified and relies on first-principle measurements (concentration and air movement measurements with well-established sensors that can be adequately calibrated).

Improved quantification of emissions from full-scale treatment steps, such as sludge storage areas, are important so that underestimations and inaccurate quantifications do not make mitigation priorities suboptimal.

Drone method discussion

Overall, the iUAS (independent uncrewed aerial system) approach appears to be powerful in its sensitivity and because all measurements are co-located in space and time (independent, having all sensors onboard), which reduces uncertainties and the need for assumptions of e.g. wind profiles and horizontal wind distribution from single point wind measurements. Highest accuracy is yielded (a) when walls jointly surrounded the target area (forming a virtual box) or (b) if using upwind and downwind walls only, when the combination of wall sizes and surroundings made the target area have a much larger impact on the net flux density change between the walls than possible contaminations from outside the target areas. In the latter case, contaminations of distributed emissions from surroundings are typically visible near wall edges and above the heights reasonably impacted by the target areas, and in such cases the flux calculations can be constrained to parts of the flight walls to maximize flux accuracy from the target area. In case (a) there is no risk of source confusion as the mass balance measures the emission of the enclosed area, and for case (b) the walls are flown, with respect to wind direction, to eliminate any source confusion in post-processing with a clear separation of plumes between sources. Overall, the ability to visually inspect gas mixing ratio, wind and GHG flux fields, in absolute or relative terms, in 2D or 3D, is highly beneficial in the data evaluation, also making it possible to find and identify unknown sources while exploring the data.

Another advantage with the iUAS method is that the very large difference in wind speeds with altitude is measured by the on-board anemometer at the exact times of the gas measurements, which would not be the case with a ground-based weather station as a supplementary instrument or

by using models based on standard 2 m or 10 m weather station placements (Figure S2). In an earlier study we have compared our iUAS method with a weather station on the ground with good agreement (Gålfalk et. al. 2021). The uncertainty calculations using a Monte-Carlo method is given in the method section. A recent study (Schultz et al. 2025) using the same drone approach have made a careful validation of the complete method by comparing with the tracer gas dispersion method.

Possible systematic uncertainty is instead associated with sensor drift in gas concentrations and wind variability over time during and between flights. The sensor drift occurs at minute frequency while the concentration variability from emission occurs at frequencies of a few seconds and were much larger than the sensor drift. The differences in temporal patterns allowed straightforward drift correction although not having any large impact on the results. When possible, repeated flights are the best way to reduce the uncertainty but for practical reasons in facilities with ongoing regulated activities and strict flight-time slots, this is not always possible. Redundancy is given by the fact that wind variability is accounted for in every point at 1 Hz in the calculations (measured at 5 Hz but resampled to 1 Hz). Hence, variability in wind speed and directions under "gusty" conditions are fully integrated with the 1 Hz calculations making the integrated net F_g represent an average across much longer time periods than the wind variability frequency, which reduces the sensitivity of our estimates to short-term wind variability, while this variability is captured by the Monte Carlo uncertainty analysis described in the Method section. We also note that the scale of the sensor drift is much smaller than the emissions at a WWTP, making the drift have negligible impact on the flux calculations.

Another type of uncertainty is due to the fact the iUAS assessments here represent short term periods (30 min to a few hours) which do not account for temporal patterns beyond the measurement time. Therefore, we primarily highlighted the results that were consistent among all or most WWTPs, while less attention is given to results from single WWTPs which would need additional work for verification.

Importantly, the iUAS approach is capable of assessing the flux density which represents a combination of both gas concentrations and wind speed. Figure S2 shows an example of a wall from point cloud of measurements to flux map. It is apparent that the wind speed (perpendicular to the wall) varies substantially, especially with altitude. This in turn can, as seen in the flux map, make parts of the wall with medium altitude and medium gas concentrations being the highest flux contributors when wind speed is considered. Similarly, high concentration parts of the wall coupled with low wind speeds and contribute lower fluxes than first expected based on a concentration map. Additional examples of flux maps in more complex environments are shown in Figures S3 and S4.

The iUAS method is a very promising approach for simultaneous measurements of different GHGs from WWTPs with high sensitivity, from point sources to extended surfaces such as full-scale sludge piles or entire WWTPs. It does, however, currently require a large drone system with expensive sensors and a high level of knowledge for mass balance calculations. There is a high potential for method development within a few years to allow smaller, less expensive, iUAS systems that are easy to fly and allow automatic emissions calculations and visualizations by the WWTP staff themselves.

References

Avfall Sverige's Development Initiative. Self-inspection of methane emissions. ISSN 1103-4092 (2019). https://www.avfallsverige.se/media/a51nivnv/egenkontroll_metanemissioner_2019_eng.pdf

Bastviken, D., Wilk, J., Duc, N.T., Gålfalk, M., Karlson, M., Neset, T.-S., Opach, T., Enrich-Prast, A. and Sundgren, I. (2022) Critical method needs in measuring greenhouse gas fluxes. Environmental Research Letters 17, 104009.

Gålfalk, M., Påledal, S.N. & Bastviken, D. Sensitive Drone Mapping of Methane Emissions without the Need for Supplementary Ground-Based Measurements. ACS Earth and Space Chemistry 5 (10), 2668-2676 (2021).

Gålfalk, M., Påledal, S.N., Sehlén, R. & Bastviken, D. Ground-based remote sensing of CH_4 and N_2O fluxes from a wastewater treatment plant and nearby biogas production with discoveries of unexpected sources. Environmental Research 204, 111978 (2022).

Moore, D.P., Li, N.P., Wendt, L.P., Castañeda, S.R., Falinski, M.M., Zhu, J-J., Song, C., Ren, Z.J., Zondlo, M.A. Underestimation of Sector-Wide Methane Emissions from United States Wastewater Treatment. Environ. Sci. Technol. 57, 4082–4090 (2023).

Scheutz, C., Knudsen, J.E., Vechi, N.T. & Knudsen, J. Validation and demonstration of a drone-based method for quantifying fugitive methane emissions. Journal of Environmental Management 373, 123467 (2025).

Song, C., Zhu, J-J., Willis, J.L., Moore, D.P., Zondlo, M.A., Ren, Z.J. Methane Emissions from Municipal Wastewater Collection and Treatment Systems. Environ. Sci. Technol. 57, 2248−2261 (2023).
